# Opioid use and perioperative pain management practices in dogs and cats in Latvia: A national survey highlighting regional disparities and generational differences

**DOI:** 10.14202/vetworld.2026.3668-3682

**Published:** 2026-08-27

**Authors:** Laura Voiko, Liga Kovalcuka, Armands Vekšins

**Affiliations:** 1 Faculty of Veterinary Medicine, Latvia University of Life Sciences and Technologies, K. Helmaņa 8, Jelgava LV-3004, Latvia.

**Keywords:** analgesia, companion animals, Latvia, multimodal analgesia, opioid analgesics, pain assessment, perioperative care, veterinary survey

## Abstract

**Background and Aim:**

Effective pain management is fundamental to animal welfare and successful perioperative care. Although multimodal analgesia incorporating opioids is considered the standard of care in companion animal practice, opioid availability and clinical use vary considerably among countries because of differences in regulations, access, and professional training. Information regarding opioid prescribing and pain management practices in the Baltic region is lacking. This study aimed to evaluate opioid availability, opioid use patterns, perioperative pain management practices, and pain assessment approaches among veterinarians in Latvia, with particular emphasis on regional disparities, surgical activity, and generational differences in clinical practice.

**Materials and Methods:**

A national cross-sectional survey was conducted using a structured online questionnaire comprising 24 multiple-choice items distributed to licensed veterinarians in Latvia between December 2023 and January 2024. The questionnaire collected information on respondent demographics, opioid availability and use, perioperative analgesic practices, pain assessment methods, adverse effects, and factors influencing opioid selection. Descriptive statistics were used to summarize responses. Associations between categorical variables were evaluated using the chi-square test, whereas relationships between professional experience and opioid availability were examined using Spearman's rank correlation and binary logistic regression.

**Results:**

A total of 101 veterinarians completed the survey, representing 10.4% of registered Latvian Veterinary Association members. Opioids were used routinely in 80.0% of practices, most commonly in combination with non-steroidal anti-inflammatory drugs as part of multimodal analgesia. Butorphanol and buprenorphine were the most widely available opioids, whereas fentanyl and methadone availability was significantly associated with higher surgical caseloads (p < 0.01). Fentanyl availability was greatest in Rīga and surrounding regions but was absent in Latgale, demonstrating marked regional disparities. Veterinarians with fewer years of professional experience reported significantly greater access to fentanyl and more frequent use of standardized pain assessment tools. Opioid selection generally reflected perceived pain severity, with butorphanol preferred for mild pain, buprenorphine for moderate pain, and fentanyl and methadone for severe pain. Despite widespread opioid adoption and positive attitudes toward analgesia, only one-third of respondents routinely used validated pain scoring systems.

**Conclusion:**

This study provides the first national overview of opioid use and perioperative pain management practices among companion animal veterinarians in Latvia and the Baltic region. The findings demonstrate broad adoption of evidence-based multimodal analgesia while identifying important regional inequalities in opioid availability and persistent underuse of standardized pain assessment tools. These results highlight priorities for continuing professional education, wider implementation of validated pain assessment methods, and improved access to essential analgesics to promote consistent, evidence-based pain management in veterinary practice.

## INTRODUCTION

Pain is an unpleasant sensory and emotional experience that significantly affects animal health and welfare [1]. In veterinary patients, pain induces neurobiological responses that may compromise physiological stability, recovery, and overall quality of life. Consequently, effective pain recognition and management are fundamental components of modern veterinary practice [2].

Pain recognition and management are particularly challenging in veterinary medicine because animals cannot verbally communicate their subjective experiences [3]. In recent decades, significant advances in pain assessment tools, species-specific pain scales, and evidence-based analgesic guidelines have established pain assessment as an essential component of clinical evaluation and the “fourth vital sign” in veterinary medicine [4–6].

Contemporary analgesic strategies emphasize multimodal approaches combining drugs with different mechanisms of action, most commonly opioids and non-steroidal anti-inflammatory drugs (NSAIDs), to improve analgesic efficacy and reduce adverse effects [7, 8].

Opioid analgesics represent a cornerstone of perioperative pain management in veterinary anesthesia owing to their potent analgesic effects, reversibility, and predictable pharmacodynamics [2, 9, 10]. However, access to opioid medications varies considerably across European countries, as regulatory restrictions and availability may limit their routine use in clinical practice [11].

Although scientific interest in veterinary pain management has increased substantially in recent years, studies continue to indicate that analgesic practices in small animal medicine remain inconsistent [12]. International surveys evaluating perioperative and chronic pain management have demonstrated increasing awareness and acceptance of analgesic principles among veterinary professionals; however, persistent gaps remain in knowledge, drug availability, and the consistent implementation of multimodal analgesic protocols [13–27].

The availability and use of opioid analgesics in veterinary medicine are influenced not only by clinical considerations but also by national regulations and market availability. Although Latvia follows European Union legislation on veterinary medicinal products, opioid procurement, storage, record-keeping, and prescription are regulated at the national level. In addition, the relatively recent introduction of methadone to the Latvian veterinary market in 2019 expanded the range of available opioid analgesics for companion animals. As a result, patterns of opioid availability and use in Latvia may not necessarily reflect those reported in Western European countries. Regional data are essential to identify country-specific trends and barriers to veterinary pain management. Although recent studies have evaluated analgesic practices and attitudes toward pain management in countries such as Slovenia, Italy, Canada, and Brazil, comparable data are currently lacking for Latvia and the Baltic region [13, 17, 21, 25]. Furthermore, none of these studies have specifically focused on opioid use patterns in small animal practice within this region despite the central role of opioids in contemporary multimodal analgesic protocols and international pain management guidelines [1, 28–32]. Consequently, factors that may influence opioid use in clinical practice, including regional differences in drug availability, professional experience, and surgical activity, remain poorly characterized.

Therefore, the primary objective of this study was to evaluate opioid availability, opioid use patterns, and perioperative pain management practices among veterinarians in Latvia. Secondary objectives were to investigate associations between opioid availability and surgical caseload, years of professional experience, geographic region, and pain assessment practices. It was hypothesized that: (1) full μ-opioid receptor agonists would be used more frequently for severe pain, whereas partial agonists would be preferred for mild-to-moderate pain; (2) practices with higher surgical caseloads would report greater availability of potent opioids such as fentanyl and methadone; and (3) veterinarians with fewer years since graduation would report greater access to potent opioids and more frequent use of standardized pain assessment tools. To the best of our knowledge, this study is the first national survey in the Baltic region to simultaneously examine associations between surgical caseload, professional experience, geographic location, and opioid selection among companion animal veterinarians. By examining these factors within a national cohort of veterinarians, this study provides a broader understanding of opioid use and perioperative pain management practices in Latvia.

## MATERIALS AND METHODS

### Ethical approval

This study was reviewed and approved by the Animal Welfare and Protection Ethics Council of the Latvia University of Life Sciences and Technologies, Jelgava, Latvia (Approval No. LLU_Dzaep_2022-5; approved on October 11, 2022). The study was conducted in accordance with the institutional ethical guidelines for research involving human participants and complied with the applicable national requirements for ethical conduct and data protection. Participation in the survey was entirely voluntary. Before accessing the questionnaire, all participants were provided with information regarding the study objectives, estimated completion time, confidentiality measures, and intended use of the collected data. Electronic informed consent was obtained from all respondents before participation. No personally identifiable information, including names, email addresses, or IP addresses, was collected. All responses were anonymized before analysis and stored in a secure, password-protected electronic database accessible only to the investigators. Participants could discontinue the survey at any time before submitting their responses without penalty. Data were analyzed and reported only in aggregate form to ensure respondent confidentiality and privacy.

### Study period and location

The survey was administered electronically across Latvia. The online questionnaire was administered electronically via Google Forms, and responses were collected between December 1, 2023, and January 1, 2024. Following survey closure, the data were exported, screened for completeness, cleaned, and statistically analyzed between January and April 2024.

### Study design

A cross-sectional online survey was conducted using a structured questionnaire. Eligible participants were licensed veterinarians practicing in Latvia. The survey was administered via Google Forms and distributed electronically by the Latvian Veterinary Association and the Latvian Veterinary Education Center through email and social media channels. One reminder was distributed during the study period to encourage participation. Only fully completed questionnaires were included. Responses were collected between December 1, 2023, and January 1, 2024. Survey responses were downloaded as an electronic spreadsheet (MS Excel for Windows). Data collection took place between December 1, 2023, and January 1, 2024.

### Data collection

Participation in the survey was voluntary, and no compensation or incentives were provided. Before beginning the questionnaire, respondents were informed about the study objectives, the estimated completion time (15–20 min), and the anonymous nature of data collection. Electronic informed consent was obtained from all participants prior to survey completion. No personally identifiable information, including names, email addresses, or IP addresses, was collected. Survey responses were stored in a password-protected electronic database accessible only to the investigators. Data were analyzed in aggregated form to preserve participant confidentiality. Respondents could review and modify their answers using a back button at the end of each page. The survey consisted of 24 multiple-choice questions.

### Questionnaire

The questionnaire was developed based on previously published surveys evaluating pain management practices and opioid use in companion animals, with adaptations to reflect local clinical practice in Latvia [10, 13, 17, 21–23, 33–35], and was not formally validated prior to distribution.

**The questionnaire consisted of six sections addressing the following domains:** demographic and professional background, availability and use of opioids in clinical practice, species-specific opioid use, indications for opioid use and practitioner perceptions, pain management practices and adverse effects, and drug selection based on pain severity. Supplementary Material 1 provides a detailed overview of the questionnaire items and response options. Item weighting was not applied; all questionnaire items contributed equally to the analysis, without adjusting for relative importance.

### Statistical analysis

Survey responses were analyzed using descriptive and inferential statistics. Frequencies and percentages were calculated for categorical variables. For multiple-response questions, each response option was analyzed independently and reported as the proportion of respondents selecting that option; therefore, percentages could exceed 100%.

Associations between categorical variables, including surgical activity, opioid availability, and pain assessment methods, were evaluated using the chi-square test of independence, with Cramer’s V calculated as a measure of effect size. Associations between ordinal variables, such as years of professional experience, and opioid availability were assessed using Spearman’s rank correlation coefficient (ρ). Binary logistic regression was performed to further evaluate the relationship between professional experience and the availability of fentanyl and methadone. Odds ratios (ORs) with 95% confidence intervals (CIs) were calculated, and model fit was assessed using Nagelkerke’s R² and the Hosmer–Lemeshow goodness-of-fit test. Statistical significance was set at p < 0.05.

Data management and descriptive analyses were performed using Microsoft Excel (Microsoft Corporation, Redmond, WA, USA), and regression analyses were conducted using Python-based statistical computations.

## RESULTS

### Response rate and sample representativeness

Of the 967 registered members of the Veterinary Association of Latvia (data provided by the National Association of Latvian Veterinarians in 2024), 101 participated in the survey, corresponding to a participation rate of 10.4%. This sample represents approximately 14.7% of the estimated 687 actively practicing veterinarians in Latvia, as reported by the Federation of Veterinarians of Europe, within a national population of approximately 1.9 million.

### Demographic and professional characteristics of respondents

A total of 101 completed questionnaires were returned by the respondents across Latvia. Most responses (57%, 57/101) were submitted by veterinarians practicing in Rīga and surrounding regions, whereas 4% (4/101) were from the Kurzeme region (Table 1).

Most participants (82.7%, n=83) reported working primarily in small animal practice. In addition, some respondents reported providing care for other species alongside their primary practice, including exotic or nontraditional companion animals (10.2%, 10/101), farm animals (10.2%, 10/101), and equine patients (4.1%, 4/101).

Most respondents (43.6%, 44/101) reported treating up to 100 animals per month, while 35.6% (36/101) treated 100–200 animals per month. The highest number of animals treated was reported in Rīga and surrounding regions, where practices managing more than 500 animals per month (6.9%, 7/101) were more frequently reported.

**Table 1:** Demographic and professional characteristics of respondents (n = 101).

**Characteristic**	**n (%)**
Region of practice	
Rīga and surrounding region	57 (56.4)
Zemgale	19 (18.8)
Vidzeme	13 (12.9)
Latgale	7 (6.9)
Kurzeme	4 (4.0)
Years of professional experience	
0–5 years	30 (28.7)
6–15 years	34 (33.7)
16–20 years	9 (7.9)
>20 years	28 (27.7)
Practice typeᵃ	
Small-animal practice	82 (81.2)
Exotic animal practice	10 (9.9)
Farm animal practice	10 (9.9)
Equine practice	4 (4.0)
Animals treated per month	
≤100	45 (43.6)
100–200	36 (35.6)
300–500	12 (11.9)
>500	8 (6.9)
Surgical activity (% of patients undergoing surgery)	
0–10%	32 (30.7)
10–20%	37 (36.6)
30–50%	28 (27.7)
>50%	4 (4.0)

ᵃPractice type = Multiple-response item; therefore, percentages do not total 100%.

### Availability of opioids

**Figure 1:**
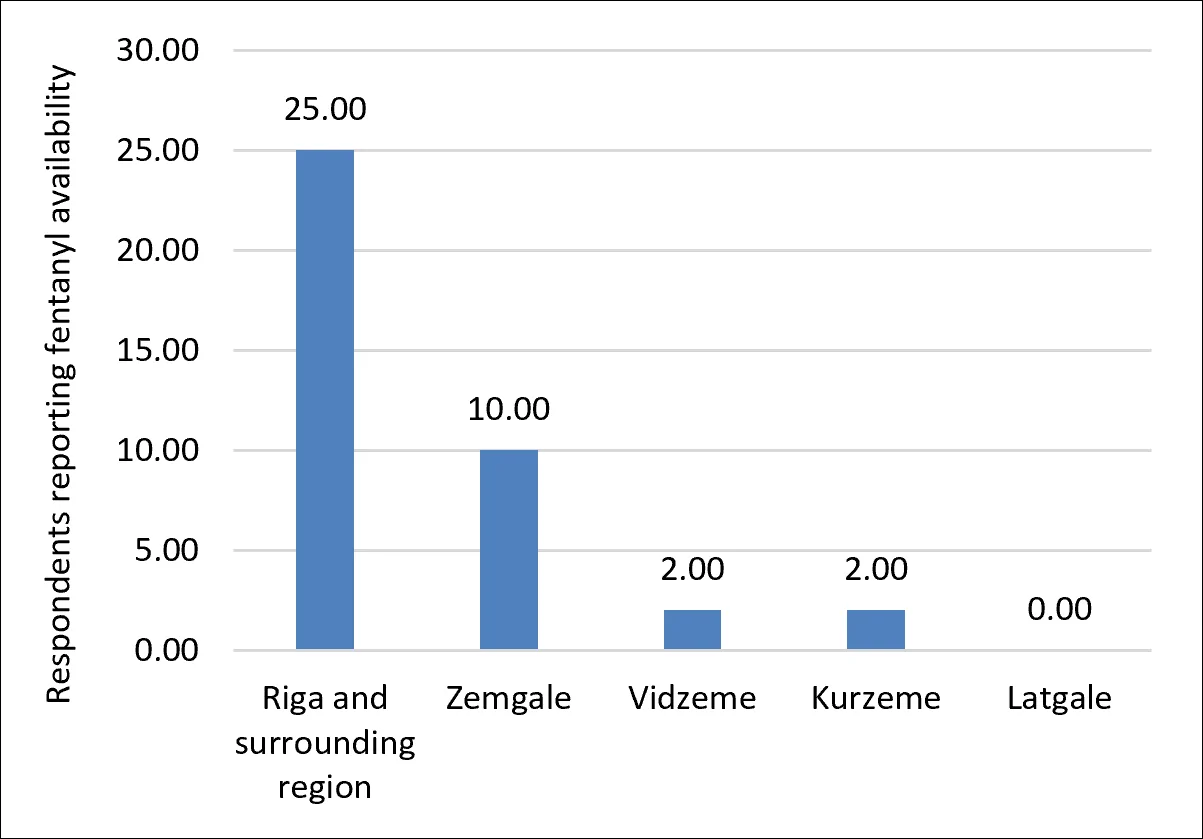
Regional distribution of fentanyl availability among surveyed veterinarians (n = 101).

Regular use of opioids in clinical practice was reported by 80% (81/101) of respondents. Among potent opioids, the μ-opioid receptor agonist methadone was reported as available by 54.1% (55/101) of respondents, followed by fentanyl (45.9%, 46/101). Fentanyl availability was reported predominantly in Rīga and surrounding regions (24.8%, 25/101), followed by Zemgale (9.9%, 10/101). Availability was considerably lower in the Vidzeme and Kurzeme regions (2% each; 2/101), whereas no availability was reported in Latgale (0/101) (Figure 1).

Morphine use was reported in only 12.9% (13/101) of the respondents’ clinical practices. The partial opioid agonist buprenorphine was reported by 83.5% (84/101) of the respondents. Among lower-potency opioids and partial μ-opioid receptor agonists, butorphanol was the most commonly available across most regions (84.7%, 86/101), while tramadol (52.9%, 53/101) showed greater prevalence in specific areas, particularly Rīga and surrounding regions.

**Figure 2:**
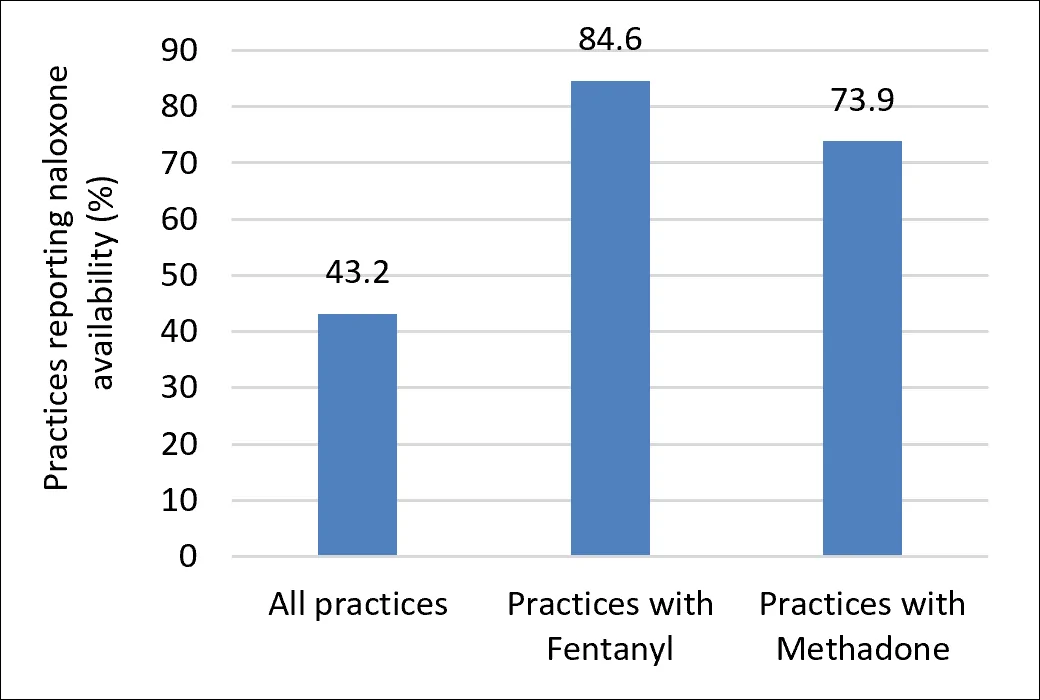
Naloxone availability among all surveyed practices and among practices reporting fentanyl or methadone availability.

Naloxone, an opioid antagonist, was available in 43.2% (44/101) of veterinary practices. Naloxone was available in 84.6% (33/39) of practices reporting fentanyl availability and 73.9% (34/46) of practices reporting methadone availability, indicating that most clinics with access to these opioids also maintained access to their antagonists (Figure 2).

### Relationship between work experience and opioid availability

**Figure 3:**
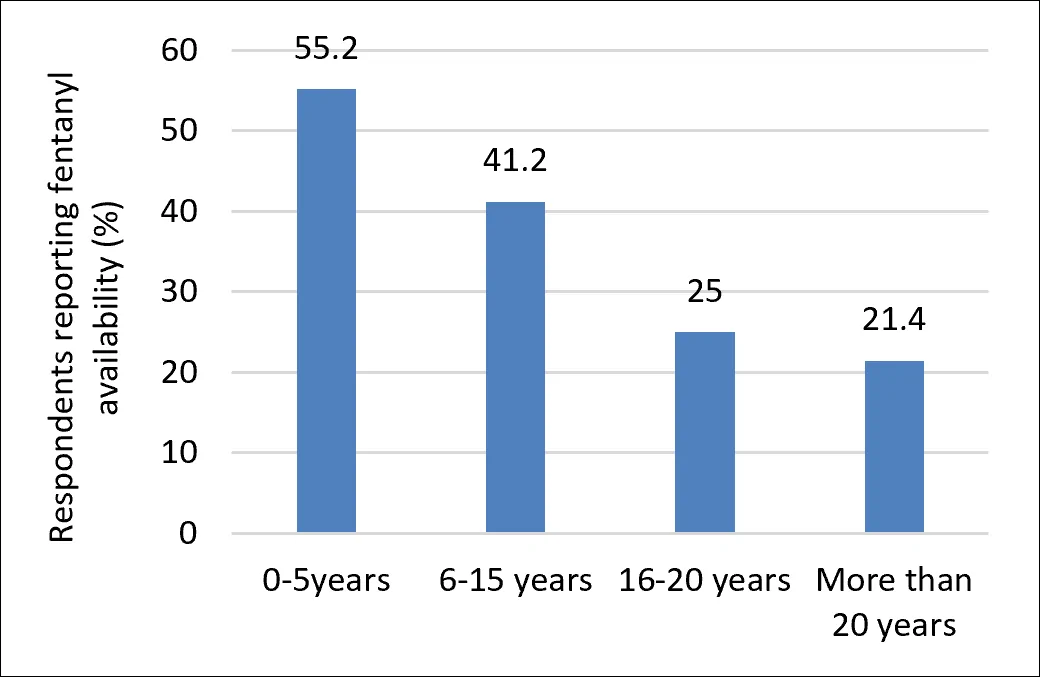
Fentanyl availability according to years of professional experience among surveyed veterinarians.

**Figure 4:**
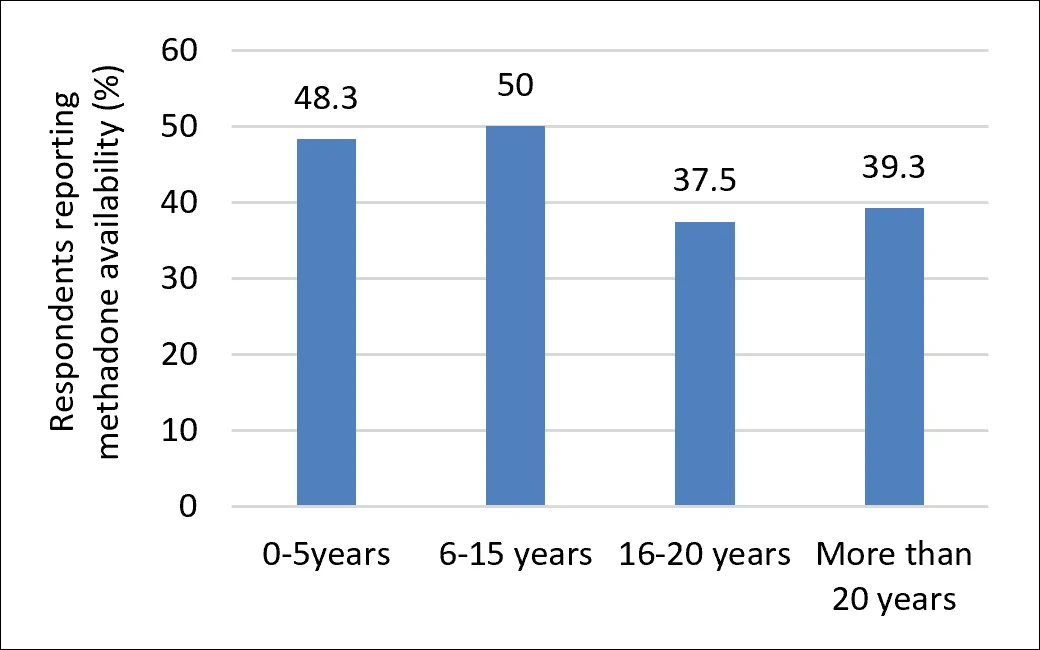
Methadone availability according to years of professional experience among surveyed veterinarians.

Most respondents were younger practitioners with <15 years of professional experience. The analysis revealed a negative association between the years of professional experience and the reported availability of fentanyl and methadone (Figures 3 and 4). Veterinarians who had graduated more recently were more likely to report access to fentanyl, whereas those with >20 years of experience reported the lowest access.

A significant negative correlation was observed between years of professional experience and fentanyl availability (Spearman’s ρ = −0.275, p = 0.0059), indicating that veterinarians with fewer years since graduation were more likely to report access to fentanyl. Logistic regression analysis confirmed this association. Increasing years of professional experience were associated with lower odds of fentanyl availability (OR=0.60, 95% CI: 0.41–0.88, p=0.008). The model demonstrated an acceptable fit (Nagelkerke R² = 0.10; Hosmer–Lemeshow p = 0.642). In contrast, no significant association was observed between work experience and methadone availability (Spearman’s ρ = −0.079, p = 0.436). Logistic regression similarly showed no significant effect of professional experience on methadone availability (OR = 0.86, 95% CI: 0.62–1.21, p = 0.400). Model fit statistics indicated limited explanatory power (Nagelkerke R²=0.01; Hosmer–Lemeshow p = 0.556).

### Association between surgical activity and opioid availability

A significant association was observed between the frequency of surgical procedures performed in veterinary practice and the availability of fentanyl and methadone. Practices in which surgical procedures accounted for 30–50% of the caseload more frequently reported the availability of these opioids compared with practices that perform fewer surgical procedures.

Chi-square analyses demonstrated a significant association between surgical activity and the availability of fentanyl (χ² = 15.39, p = 0.0015). The effect size was moderate (Cramer’s V=0.39), indicating that practices with higher surgical caseloads were more likely to report fentanyl availability. A significant association was also observed between surgical activity and methadone availability (χ² (3) = 17.50, p = 0.0006). The effect size was moderate (Cramer’s V = 0.42), suggesting greater methadone availability in practices that perform a higher proportion of surgical procedures.

Analysis of responses concerning opioid use in surgical procedures revealed that the most frequently reported challenges were associated with human error and record-keeping (62.4%, 63/101), as well as legal and documentation requirements (40.6%, 41/101). Less frequently, respondents cited difficulties with drug availability or procurement (13.9%, 14/101) and storage requirements (12.9%, 13/101).

### Species-specific opioid use

The most frequently reported opioids were buprenorphine (42.0%, 42/101) and butorphanol (32.1%, 32/101), followed by methadone (19.8%, 20/101). Analysis of responses to Question 12 revealed distinct patterns of opioid use across species. Fentanyl (21.8%, 22/101) and tramadol (15.8%, 16/101) were most frequently reported in dogs, whereas butorphanol (21.8%, 22/101) and buprenorphine (22.8%, 23/101) were commonly reported in both dogs and cats.

Methadone was reported in both dogs and cats, with higher reporting rates in dogs (14.9%, 15/101) and in combined dog–cat practices (16.8%, 17/101). In contrast, morphine was rarely used, with most respondents (7.9%, 8/101) indicating that it was not used.

### Indications for opioid use and perceptions

Veterinarians reported using opioids for various clinical purposes, with therapeutic pain management (47.6%, 48/101), intraoperative analgesia (45.2%, 46/101), and postoperative pain control (33.3%, 34/101) being the most frequently selected indications. In addition, 21.4% (22/101) of respondents reported using opioids in palliative care settings.

When asked about the surgical procedures during which opioids were administered, 55.1% (56/101) of respondents indicated their use during all listed interventions. Among the individual procedures, opioids were most frequently reported for soft tissue surgeries (43.6%, 44/101) and castration or ovariohysterectomy (42.3%, 43/101), followed by dental and oral procedures (35.9%, 36/101) and orthopedic surgeries (21.8%, 22/101).

Tramadol use varied across practitioners with different levels of surgical activity. It was most frequently reported in practices where 10–20% of registered animals underwent surgery (34.4%, 11/101), followed by 30–50% (28.1%, 9 respondents), and 0–10% (25.0%, 8 respondents). Only a small proportion (6.3%, 2 respondents) of practices involving surgical caseloads exceeding 50% reported tramadol use.

A positive assessment of the introduction of opioids into veterinary practice was reported by 96.6% (98/101) of respondents. In total, 62.4% (63/101) of respondents evaluated their knowledge of pain assessment and opioid use as adequate, but in need of improvement.

**Figure 5:**
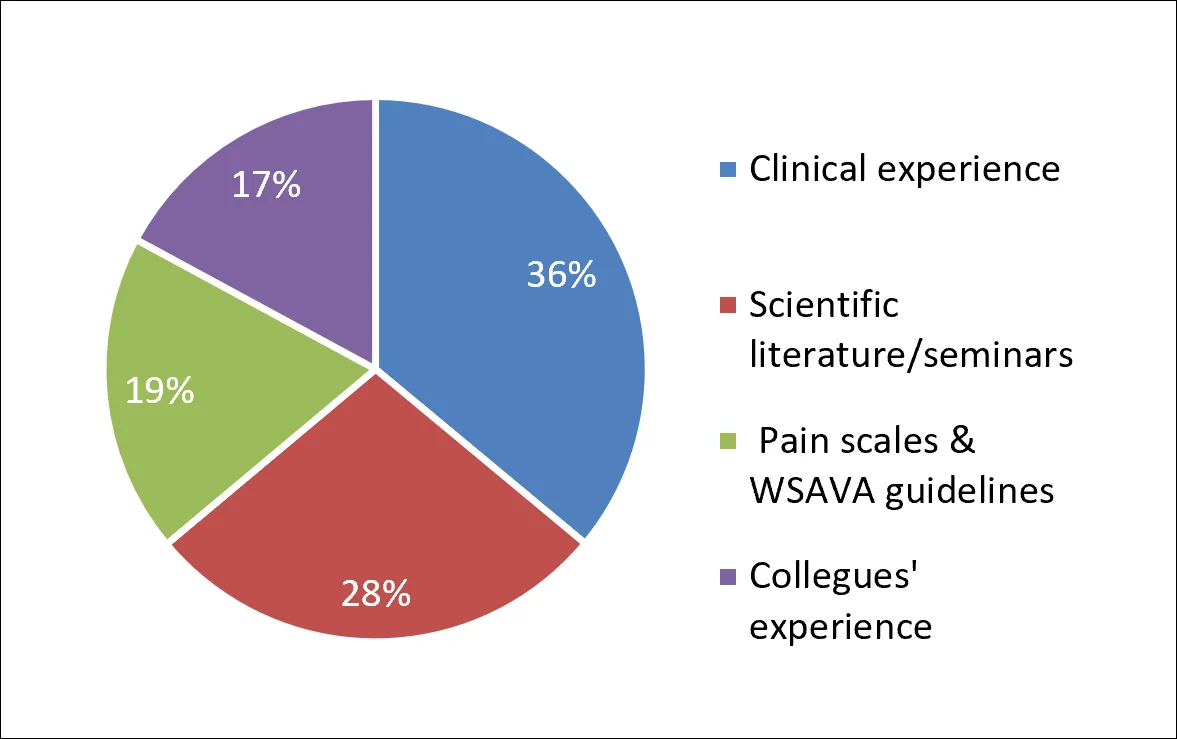
Methods used for pain assessment in animals.

When asked how pain is assessed in animals, 62.6% (62/99) of respondents reported relying primarily on their own clinical experiences. In addition, 48.4% (46/95) reported consulting scientific literature or knowledge obtained from seminars and conferences, 33.0% (33/100) reported using pain scales and the World Small Animal Veterinary Association (WSAVA) pain assessment guidelines, and 29.7% (30/101) relied on their colleagues’ experiences. Respondents were able to select more than one option (Figure 5).

A chi-square test of independence demonstrated a significant association between years of professional experience and pain assessment methods (χ² = 27.18, df = 12, p = 0.0073; Cramer’s V = 0.303). Among veterinarians with 0–5 years of professional experience, 41% reported using formal pain scales and the WSAVA guidelines, whereas 50% of those with >20 years of experience reported relying primarily on personal clinical experience.

### Pain management practices and adverse effects

Sedation was the most common adverse effect (61.0%, 62/101) observed in conscious animals. Other reported adverse effects included hypersalivation (41.5%, 42/101), panting (37.8%, 38/101), and dysphoria characterized by vocalizations and restlessness (36.6%, 37/101). Bradycardia and hypotension were reported by 23.2% (23/101) of respondents, while vomiting was reported by 3.7% (4/101). When opioids were administered during anesthesia, bradycardia was reported by 58.9% (59/101) of respondents. Hypotension was reported in 35.6% (36/101) of cases, while hypothermia and respiratory depression were reported by 28.8% (29/101). Table 2 provides a detailed overview of the adverse effects associated with opioid administration. Opioids were combined with NSAIDs by 51.1% (52/101) of respondents, primarily for the management of acute pain.

### Drug selection for pain based on severity

Butorphanol (29.7%, 30/101) and buprenorphine (14.9%, 15/101) were the most commonly reported opioids for mild pain. Tramadol (5.9%, 6/101) and methadone (2.0%, 2/101) were reported less frequently. In cases of moderate pain, buprenorphine was reported by 23.8% (24/101) of respondents, followed by butorphanol (11.9%, 12/101) and methadone (10.9%, 11/101). Fentanyl (39.6%, 40/101) and methadone (31.7%, 32/101) were the most frequently reported opioids used to treat severe pain. Buprenorphine was reported by 26.7% (27/101) of respondents, while morphine was reported by 10.9% (11/101). Table 3 provides a detailed overview of the opioid selection according to perceived pain severity.

**Table 2:** Reported adverse effects associated with opioid administration in small animals under different clinical conditions.

**Clinical context**	**Adverse effect**	**Respondents reporting adverse effect, n (%)**
Conscious animals	Sedation	62 (61.0)
	Hypersalivation	42 (41.5)
	Panting	38 (37.8)
	Dysphoria (vocalization, restlessness)	37 (36.6)
	Bradycardia and hypotension	23 (23.2)
	Vomiting	4 (3.7)
During anesthesia	Bradycardia	59 (58.9)
	Hypotension	36 (35.6)
	Hypothermia	29 (28.8)
	Respiratory depression	29 (28.8)

Values = Number (percentage) of respondents (n = 101) reporting each adverse effect. Respondents could select multiple adverse effects; therefore, percentages within each clinical context may exceed 100%. Adverse effects are presented separately for conscious animals and during anesthesia to reflect differences in clinical context and monitoring conditions.

**Table 3:** Opioid selection according to perceived pain severity in dogs and cats among surveyed veterinarians.

**Pain severity**	**Opioid**	**Respondents selecting opioid, n (%)**
Mild pain	Butorphanol	30 (29.7)
	Buprenorphine	15 (14.9)
	Tramadol	6 (5.9)
	Methadone	2 (2.0)
Moderate pain	Buprenorphine	24 (23.8)
	Butorphanol	12 (11.9)
	Methadone	11 (10.9)
Severe pain	Fentanyl	40 (39.6)
	Methadone	32 (31.7)
	Buprenorphine	27 (26.7)
	Morphine	11 (10.9)

Values = Number (percentage) of respondents (n = 101) selecting each opioid for the indicated pain severity category. Respondents could select more than one opioid within each pain severity category; therefore, percentages may exceed 100%. Pain severity categories (mild, moderate, and severe) were based on respondents' subjective clinical assessment and were not standardized across participants. Reported opioid selections reflect drug availability, clinical preference, and individual experience rather than standardized treatment protocols.

## DISCUSSION

### Overview of findings

This study evaluated the patterns of opioid use and pain assessment practices among veterinarians in Latvia, based on data obtained from 101 respondents. To the best of our knowledge, this is the first systematic investigation focusing specifically on attitudes toward opioid use, pain perception, and analgesic strategies in small animal practices in Latvia. Although the response rate was modest (10.4%), the demographic and professional distribution of the participants suggests that the findings provide meaningful insights into the current clinical practice.

Several findings warrant particular attention. First, substantial regional differences were observed in the availability of potent opioids, with fentanyl and methadone reported predominantly in Rīga and surrounding regions and considerably less frequently in other parts of the country. Second, years of professional experience were associated with both fentanyl availability and pain assessment practices, suggesting generational differences in the adoption of evidence-based analgesic approaches. Third, methadone was reported as available by more than half of respondents despite its relatively recent introduction to the Latvian veterinary market, indicating rapid integration into clinical practice. Together, these findings provide important insights into factors influencing veterinary pain management in Latvia.

Although no recent studies have exclusively examined opioid use in small veterinary practices in Latvia, comparable trends can be inferred from international surveys that evaluate veterinarians’ approaches to pain management in companion animals. Collectively, these investigations indicate that opioid utilization is influenced by clinician perceptions of pain, regulatory constraints, drug availability, and the overall emphasis placed on analgesia within individual practices [17, 21, 27, 36].

### Opioid selection according to pain severity

Opioid selection among Latvian veterinarians reflects appropriate differentiation according to pain severity. Regarding severe pain, fentanyl (39.6%, 40/101) and methadone (31.7%, 32/101) were the most frequently reported opioids, whereas buprenorphine (26.7%, 27/101) and morphine (10.9%, 11/101) were reported less frequently. These findings indicate that opioid selection generally aligned with contemporary analgesic principles and international recommendations for perioperative analgesia, with butorphanol most commonly reported for mild pain, buprenorphine for moderate pain, and fentanyl and methadone for severe pain [37, 38].

These findings support our first hypothesis that full μ-opioid receptor agonists would be selected more frequently for severe pain, whereas partial agonists would be preferred for mild-to-moderate pain.

### Opioid availability and regional variation

The introduction of opioids into veterinary practice was overwhelmingly perceived as beneficial, with 96.6% (98/101) of respondents reporting improvements in analgesia and animal welfare. Only a small proportion of the veterinarians considered opioid availability to have a negative or no impact. This strong professional consensus reinforces the role of opioids as a central component of effective pain management and aligns with international recommendations advocating their use in multimodal analgesic protocols.

Opioids are a fundamental component of acute pain management in Latvian veterinary practice, consistent with findings from other countries [21, 27]. However, their availability markedly varies across regions and practice profiles. Fentanyl and methadone were significantly more likely to be available in clinics with higher surgical caseloads (≥ 30% of registered animals), highlighting the close relationship between surgical activity and the procurement of potent opioids. This observation is consistent with international evidence demonstrating that access to μ-opioid receptor agonists is greatest in practices with substantial perioperative analgesic demand [37, 39].

**Geographical disparities were pronounced:** fentanyl and methadone were predominantly available in Rīga and the surrounding regions, followed by Zemgale, while availability was minimal in Vidzeme and Kurzeme and absent in Latgale. These patterns likely reflect referral structures and the concentration of complex surgical procedures in urban centers. Although less likely, it cannot be ruled out that anesthetic standards and analgesic practices may differ in more rural settings, warranting further investigation. The concentration of small animal veterinary practices in Rīga and the surrounding regions may further contribute to these observed differences.

Several factors may contribute to these regional differences. Veterinary referral centers and practices performing advanced surgical procedures are concentrated primarily in larger urban areas (Rīga and Jelgava), which may increase demand for potent opioids and justify the infrastructure required for their storage and administration. Economic considerations may also influence purchasing decisions, particularly in smaller clinics with lower surgical caseloads. In addition, compliance with controlled-drug regulations requires administrative and record-keeping procedures that may represent a greater burden for smaller practices. Although these explanations remain speculative, the findings suggest that access to potent analgesics may not be uniform throughout the country.

Morphine availability was notably low, reported by only 12.9% of respondents, in contrast to earlier surveys from New Zealand, in which morphine remained the most commonly used opioid [27]. This discrepancy may be partly explained by the introduction of methadone to the Latvian veterinary market in 2019, which led to a gradual shift in prescription preferences. Similar transitions have been reported in Australia, Slovenia, Italy, and Switzerland, where methadone, buprenorphine, and butorphanol have emerged as the preferred opioids in small animal practice [9, 16, 22, 25].

The widespread use of butorphanol and buprenorphine among Latvian veterinarians is consistent with international trends and likely reflects their favorable safety profiles, licensing status, and historical availability. In Latvia, butorphanol was not classified as a controlled substance for an extended period, which facilitates its routine use. Partial agonists are generally associated with a lower risk of respiratory depression and bradycardia compared with pure μ-opioid agonists, which may further explain their popularity [40, 41]. Additionally, the ease of intramuscular administration and longer analgesic duration of buprenorphine contribute to its clinical appeal [42, 43].

The high proportion of clinics reporting concurrent access to naloxone indicates appropriate awareness of opioid safety and preparedness for adverse effects. Naloxone availability was reported by most practices stocking fentanyl or methadone, reflecting responsible opioid stewardship in these settings.

### Demographic and clinical determinants of opioid use

Most respondents were younger practitioners with <15 years of professional experience, a demographic profile comparable to that of surveys conducted in Australia and Thailand [9, 26]. The observed negative association between years of professional experience and fentanyl availability suggests a generational shift in analgesic practice. This trend may reflect the increased exposure to opioid pharmacology, multimodal analgesia, and evidence-based pain management during veterinary training over the past decade.

Veterinarians with fewer years of professional experience are more likely to report access to fentanyl, potentially reflecting curricular changes and greater engagement in continuing education [44]. Over the past decade, veterinary curricula have increasingly emphasized anesthesiology, analgesia, and evidence-based pain management, which may contribute to greater familiarity and confidence in the use of potent opioids among more recently trained practitioners. In contrast, methadone availability did not show a similar association, suggesting that its use may be influenced more strongly by regulatory and logistic factors than by generational training alone. The requirements for infusion pumps and advanced monitoring equipment may further limit the use of fentanyl in smaller- or lower-volume clinics.

The strong association between surgical caseload and opioid availability reinforces the role of clinical demand in shaping analgesic practices. Clinics that perform a high proportion of surgical procedures are significantly more likely to stock fentanyl and methadone, supporting the central role of these agents in perioperative pain management [45]. Conversely, practices with lower surgical volumes may underprioritize investment in controlled analgesics, potentially contributing to disparities in pain management standards.

### Species-specific preferences and surgical context

Clear species-related patterns of opioid selection were identified. Fentanyl and tramadol were predominantly used in dogs, whereas butorphanol and buprenorphine were more evenly distributed among canine and feline patients. Morphine use was infrequent, consistent with international reports of declining clinical preference and limited availability in small-animal practice.

Tramadol use varied according to surgical activity, with a higher prevalence in clinics with moderate surgical caseloads and reduced use in high-volume surgical practices. This pattern may reflect differences in clinicians’ familiarity with, perceived efficacy of, and access to alternative analgesics. However, evidence supporting the efficacy of tramadol in dogs remains inconsistent. Experimental and clinical studies have indicated limited intraoperative analgesic effects, whereas modest postoperative benefits have been reported, particularly when tramadol is used as part of multimodal protocols [46, 47].

In contrast, tramadol appears to provide consistent postoperative analgesia in cats. Randomized clinical trials have demonstrated effective pain control following routine surgery, especially when combined with NSAIDs [48, 49]. These findings highlight the species-specific nature of the analgesic utility of tramadol and underscore the importance of tailoring analgesic protocols.

The observed association between surgical activity and fentanyl and methadone availability supports our second hypothesis that practices with higher surgical caseloads would report greater access to potent opioids.

### Pain assessment practices and generational differences

Differences in pain assessment approaches were evident across generations. Veterinarians who had graduated more recently were more likely to use structured tools, such as validated pain scales and the WSAVA guidelines, whereas more experienced clinicians predominantly relied on clinical judgment. Although the strength of this association was modest, it mirrors the findings of multiple international surveys [9, 50, 51].

Barriers to routine pain scoring, including time constraints, limited training, and the perceived rigidity of assessment tools, have been consistently reported in the literature [29, 52]. Nevertheless, the increasing use of evidence-based resources among younger practitioners suggests a gradual shift toward standardized pain assessment, with continued education playing a crucial role in facilitating this transition.

The increased use of pain scales and greater reported fentanyl availability among less experienced veterinarians are consistent with our third hypothesis regarding generational differences in analgesic practices.

The observed generational differences in pain assessment practices may reflect changes in veterinary education and increased emphasis on evidence-based pain management during recent decades. Nevertheless, only one-third of respondents reported using formal pain scales, indicating considerable scope for improvement. Continuing education initiatives focusing on pain recognition, implementation of validated pain assessment tools, and multimodal analgesic strategies may facilitate broader adoption of standardized approaches to pain management in clinical practice.

### Adverse effects and perceptions of opioid use

The reported adverse effects were consistent with known opioid pharmacodynamics. Sedation was the most frequently observed effect in the conscious animals, followed by hypersalivation, panting, and dysphoria. Cardiovascular effects, including bradycardia and hypotension, have been reported less frequently, whereas respiratory depression is uncommon. These findings are consistent with those of previous studies and emphasize the importance of vigilant monitoring during opioid administration, especially under anesthesia [38, 53].

Misconceptions regarding opioid safety persist. Several surveys have demonstrated a limited awareness of species-specific adverse effects that may contribute to hesitation in opioid use [20, 54]. These observations underscore the need for targeted education to improve our understanding of opioid pharmacology and promote confident evidence-based analgesic use.

### Clinical implications

Overall, opioid selection among Latvian veterinarians reflected appropriate differentiation according to pain severity, with partial agonists predominantly used for mild-to-moderate pain and potent μ-opioid agonists reserved for severe pain. The widespread concurrent use of NSAIDs further supports the adoption of multimodal analgesic strategies, which is consistent with international guidelines. Taken together, these findings suggest that, while opioid use and pain management practices in Latvia generally align with evidence-based principles, disparities in access, training, and pain assessment remain. Addressing these gaps through continued education, policy initiatives, and improved access to analgesic resources may help promote uniform standards of pain management across veterinary practices.

From an animal welfare perspective, unequal access to potent opioid analgesics may contribute to variation in pain management options available to veterinary patients across regions. Although opioids represent only one component of multimodal analgesia, limited access to full μ-opioid receptor agonists may reduce the flexibility of analgesic protocols for patients undergoing major surgical procedures or experiencing severe pain. Therefore, continued efforts to improve access to evidence-based pain management strategies have the potential to enhance animal welfare outcomes at a national level.

The findings of this study highlight several opportunities to strengthen veterinary pain management in Latvia. First, wider implementation of validated pain assessment tools should be encouraged through undergraduate education and continuing professional development programs. Second, dissemination of national or international pain management guidelines may help promote more consistent analgesic practices across regions. Finally, further evaluation of factors influencing opioid availability, particularly in regions reporting limited access to fentanyl and methadone, may help identify practical or regulatory barriers affecting the provision of perioperative analgesia.

### Limitations

Several limitations should be considered when interpreting the findings of this study. First, the response rate was relatively modest (10.4%), and participation was voluntary, introducing the possibility of selection bias. Veterinarians with a particular interest in anesthesia, analgesia, or pain management may have been more likely to participate, potentially resulting in an overestimation of opioid availability, opioid use, and evidence-based pain management practices. In addition, respondents from Rīga and the surrounding regions were overrepresented compared with those from other geographic regions. Formal assessment of non-response bias was not possible because demographic information regarding non-respondents was unavailable. Furthermore, the survey was distributed electronically via email and social media, and not all veterinarians may routinely use these communication channels.

Second, the study relied on self-reported data and was therefore subject to social desirability and recall bias. Respondents may have reported practices perceived as professionally desirable, particularly regarding pain assessment and opioid use, while recollection of adverse effects and prescribing practices may have been imperfect. No objective measures, such as prescription records, controlled-drug inventories, or patient case logs, were available for verification; therefore, the findings reflect reported rather than directly observed clinical practice. In addition, patient-level outcomes, analgesic efficacy, and welfare indicators were not evaluated.

Finally, the questionnaire was adapted from previously published surveys but was not formally validated before distribution. It consisted exclusively of closed-ended questions and did not include open-ended qualitative items, preventing mixed-methods analysis. Furthermore, information regarding opioid dosing regimens, routes of administration, fentanyl constant rate infusion versus bolus administration, treatment duration, and the use of local or regional anesthesia was not collected. Despite these limitations, this study provides the first national overview of opioid availability, opioid use patterns, and pain assessment practices among veterinarians in Latvia and identifies important regional and generational trends that warrant further investigation.

## CONCLUSION

This study evaluated opioid use and perioperative pain management practices among veterinarians in Latvia through a national survey of 101 respondents. *K*ey results showed high adoption of opioids (80% daily use), with buprenorphine and butorphanol most available and fentanyl/methadone more common in high-surgical-volume practices in Rīga and surrounding regions. Significant generational differences emerged, with younger veterinarians reporting greater fentanyl availability and more frequent use of standardized pain scales. Regional disparities were pronounced, and multimodal analgesia (especially with NSAIDs) was common, though formal pain scoring remained underutilized (33%).

These findings highlight the need for targeted interventions to reduce regional and generational gaps in opioid access and standardized pain assessment. Improved availability of potent opioids in rural areas, combined with continuing education on evidence-based protocols, could enhance animal welfare and align Latvian practices more closely with international guidelines.

This is the first national survey in the Baltic region to examine opioid patterns alongside surgical caseload, professional experience, and pain assessment practices, providing valuable country-specific baseline data.

The modest response rate (10.4%) and potential selection bias toward interested respondents, overrepresentation of urban practitioners, reliance on self-reported data, and lack of validation of the questionnaire limit generalizability. Patient outcomes and objective measures (e.g., prescription records) were not assessed.

Larger studies with higher response rates, objective verification of practices, inclusion of qualitative insights, and evaluation of patient-level outcomes are warranted. Longitudinal assessments following educational interventions and policy changes regarding opioid access could further inform improvements in veterinary pain management across Latvia and the broader Baltic region.

Opioid selection in Latvia generally aligned with contemporary analgesic principles, with clear differentiation by pain severity and widespread multimodal use. However, marked regional disparities, generational differences in adoption of best practices, and underutilization of standardized pain assessment tools indicate persistent gaps. Addressing these through education, improved access, and policy support will be essential to ensure consistent, high-quality perioperative pain management and better animal welfare outcomes nationwide.

## DATA AVAILABILITY

The supplementary data are available from the corresponding author upon reasonable request.

## GENERATIVE AI DECLARATION

The authors used OpenAI to assist with language editing and to improve the clarity and structure of the manuscript. All AI-assisted content was critically reviewed, revised, and verified by the authors, who take full responsibility for the accuracy, originality, and integrity of the final manuscript.

## AUTHORS’ CONTRIBUTIONS

LV: Conceived and designed the study, developed the questionnaire, collected and analyzed the data, performed the statistical analyses, interpreted the findings, and drafted the manuscript. AV and LK: Contributed to the study design, interpreted the results, and critically revised the manuscript. All authors have read and approved the final manuscript.
